# Ntrk1 promotes mesangial cell proliferation and inflammation in rat glomerulonephritis model by activating the STAT3 and p38/ERK MAPK signaling pathways

**DOI:** 10.1186/s12882-022-03001-4

**Published:** 2022-12-28

**Authors:** Xiongjun Dong, Yingchun Tang

**Affiliations:** Blood Purification Center, The Second People’s Hospital of Wuhu, Anhui Province, 241000 China

**Keywords:** MsPGN, Ntrk1, Glomerulus, Mesangial cel, Proliferation, Inflammation

## Abstract

**Background:**

Mesangial proliferative glomerulonephritis (MsPGN) accounts for a main cause of chronic kidney disease (CKD), chronic renal failure and uremia. This paper aimed to examine the effect of Ntrk1 on MsPGN development, so as to identify a novel therapeutic target for MsPGN.

**Methods:**

The MsPGN rat model was constructed by single injection of Thy1.1 monoclonal antibody via the tail vein. Additionally, the Ntrk1 knockdown rat model was established by injection of Ntrk1-RNAi lentivirus via the tail vein. Periodic acid-schiff staining and immunohistochemistry (IHC) were performed on kidney tissues. Moreover, the rat urinary protein was detected. Mesangial cells were transfected and treated with p38 inhibitor (SB202190) and ERK inhibitor (PD98059). Meanwhile, the viability and proliferation of mesangial cells were analyzed by cell counting kit-8 (CCK-8) and 5-Ethynyl-2′-deoxyuridine assays. Gene expression was detected by quantitative real-time polymerase chain reaction (qRT-PCR) and Western-blot (WB) assays.

**Results:**

The proliferation of mesangial cells was enhanced in glomerulus and Ki67 expression was up-regulated in renal tubule of MsPGN rats. The urine protein level increased in MsPGN rats. Pro-inflammatory factors and Ntrk1 expression were up-regulated in glomerulus of MsPGN rats. Ntrk1 up-regulation promoted the viability, proliferation, expression of pro-inflammatory factors and activation of the STAT3, p38 and ERK signaling pathways in mesangial cells. Ntrk1 knockdown reduced mesangial cell proliferation, urine protein, pro-inflammatory factors, activation of STAT3, p38 and ERK signaling pathways in glomerulus, and decreased Ki67 expression in renal tubule of MsPGN rats. Treatment with SB202190 and PD98059 reversed the effect of Ntrk1 on promoting the viability, proliferation and inflammatory response of mesangial cells.

**Conclusion:**

Ntrk1 promoted mesangial cell proliferation and inflammation in MsPGN rats by activating the STAT3 and p38/ERK MAPK signaling pathways.

**Supplementary Information:**

The online version contains supplementary material available at 10.1186/s12882-022-03001-4.

## Background

Glomerulonephritis is one of the main causes of end-stage renal disease (ESRD) all over the world. The incidence of glomerulonephritis ranges from 10.5% to 38.2% and its prevalence is 17.6%-53.5% [1]. Mesangial proliferative glomerulonephritis (MsPGN) is characterized by the increased number of cells and extracellular matrix (ECM) in the mesangial region of glomerulus [2]. The proliferation of mesangial cells can release inflammatory mediators to induce interstitial fibrosis and irreversible progressive glomerulosclerosis, eventually evolving into ESRD [3]. The proliferation and inflammation of mesangial cells play an important role in the development of MsPGN [4]. Therefore, reducing the proliferation and inflammation of mesangial cells is of great significance for improving the therapeutic effect of MsPGN. In recent years, research on the molecular mechanisms of human diseases has provided more potential therapeutic targets [5, 6]. Exploring the molecular mechanism of MsPGN development is conductive to finding effective targets for alleviating the proliferation and inflammation of mesangial cells.

Neurotrophic tropomyosin receptor kinase 1 (Ntrk1), located at 1q21-q22, belongs to the tyrosine receptor kinase (Trk) family. The protein encoded by Ntrk1 is also called tyrosine receptor kinase A (TrkA) [7]. Previous study has discovered that TrkA expression is up-regulated in kidney tissues of patients with diabetic nephropathy (DN). However, in healthy human kidney tissues, TrkA is only expressed at a low level. Animal experiment indicates that inhibition of TrkA may be conductive to preventing kidney fibrosis [8]. Bonofiglio et al*.* [9] discovered that in kidney biopsy samples from patients with renal diseases, TrkA expression was detected in tubular and glomerular cells. In addition, TrkA was over-expressed in the peripheral blood mononuclear cells (PMNCs) in glomerulonephritis patients [10]. However, the exact role of Ntrk1 in MsPGN and the underlying mechanism have not been elucidated yet. Thus, in this study, the MsPGN rat model was constructed. The exact expression and effect of Ntrk1 on the proliferation and inflammation of mesangial cells were investigated. This article can provide a novel therapeutic target for MsPGN.

## Methods

### Ethics committee

The animal experiment in this study was approved by the Animal Ethics Committee of The Second People's Hospital of Wuhu.

### Construction of the MsPGN rat model

Twelve male SD rats weighing 200–220 g were purchased from Shanghai Laboratory Animal Center, Chinese Academy of Sciences. All rats had free access to food and water, and were kept in a room under 22 °C and 12-h/12-h light/dark cycle conditions. Nine rats were used to construct the MsPGN model by single injection of Thy1.1 monoclonal antibody (2.5 mg/kg, ab95812, Abcam, Shanghai, China) via the tail vein [11]. The Thy1.1 monoclonal antibody was generated by OX-7 cells. At 3, 7 and 14 days after injection, three rats were euthanized to obtain kidney tissues. The glomeruli were isolated as described previously [12]. Furthermore, the other three rats were given injection of an equivalent volume of normal saline and then euthanized on day 0 to collect kidney tissues and glomeruli. The kidney tissues and glomeruli were later stored at -80 °C.

Rats were euthanized as follows: Rats were deeply anesthetized by inhalation of 5% isoflurane [13]. When rats had no response to head and limb stimulation, they were rapidly executed by cervical dislocation. After 10 s of cervical dislocation, rats were identified as dead if they stopped breathing and had no respond to systemic stimulation [14].

### Construction of the Ntrk1 knockdown rat model

Male SD rats weighing 200–220 g (*n* = 36, Shanghai Laboratory Animal Center, Chinese Academy of Sciences) were randomly divided into three groups, including PBS group (*n* = 12), shNC group (*n* = 12) and shNtrk1 group (*n* = 12). Rats of each group had free access to water and food and raised in a room under 22 °C and 12-h/12-h light/dark cycle conditions. Rats of PBS group, shNC group and shNtrk1 group were respectively injected with 0.2 mL phosphate buffered solution (PBS), 0.2 mL negative control lentivirus (1 × 10^9^ TU/mL) and Ntrk1-RNAi lentivirus (1 × 10^9^ TU/mL) via tail vein thrice at 5-day intervals [15]. Afterwards, three rats from each group were randomly selected for euthanasia on days 0, 3, 7 and 14. The kidney tissues were obtained, the glomeruli were isolated, and all of them were stored at -80 °C.

Male SD rats weighing 200–220 g (*n* = 9, Shanghai Laboratory Animal Center, Chinese Academy of Sciences) were randomly divided into three groups, namely, Sham group (*n* = 3), shNC + Thy-1 GN group (*n* = 3) and shNtrk1 + Thy-1 GN group (*n* = 3). All rats had free access to water and food, and were raised in a room under 22 °C and 12-h/12-h light/dark cycle conditions. Rats of Sham group did not receive any treatment, while those of shNC + Thy-1 GN group and shNtrk1 + Thy-1 GN group were given injection of negative control lentivirus (1 × 10^9^ TU/mL) and Ntrk1-RNAi lentivirus (1 × 10^9^ TU/mL), respectively, via the tail vein thrice at 5-days intervals. Thereafter, rats from the two groups were subject to injection of Thy1.1 monoclonal antibody (2.5 mg/kg) via the tail vein. On day 7 of Thy1.1 monoclonal antibody injection, rats of each group were euthanized. Afterwards, kidney tissues were obtained, glomeruli were isolated, and all of them were stored at -80 °C.

### Periodic acid-schiff (PAS) staining

The rat kidney tissues were fixed with 10% formaldehyde solution, embedded in paraffin, and prepared into 4-μm sections. After xylene deparaffinage, the sections were rehydrated with gradient ethanol and stained with PAS. Hematoxylin was later utilized for the counter-staining of sections. The total cell number in each glomerulus cross-section was counted under a microscope. For each rat, cells were counted in three consecutive sections of 10 glomeruli. Simultaneously, the mesangial matrix score was evaluated to determine the glomerulus damage degree. The scoring standard was as follows: 0 point, normal; 1 point, slight glomerulus damage of mesangial matrix and hyalinosis with focal adhesion involving < 25% of the glomerulus; 2 points, 25%-50% of glomerulus sclerosis; 3 points, 50%-75% of sclerosis; and 4 points, > 75% of sclerosis [16]. The experiment was independently repeated thrice.

### Immunohistochemistry (IHC) analysis

The 4-μm kidney tissue sections were deparaffinized with xylene, rehydrated with an ascending series of gradient ethanol, and blocked with 3% H_2_O_2_. Thereafter, the sections were incubated with sodium citrate buffer (pH = 6.0) for 10 min and later with goat serum (5%) for 20 min. After washing, the sections were incubated with rabbit anti-Ki67 primary antibody (1:100, ab92742, Abcam, Cambridge, MA, USA) for 12 h at 4 °C. Afterwards, the unbound primary antibody was washed with PBS, and the sections were further incubated with biotinylated secondary antibody for 40 min at room temperature. Thereafter, sections were treated with horseradish peroxidase (HRP)-streptavidin working buffer for 40 min at room temperature, and later stained with 3,3'-diaminobenzidine (DAB) and hematoxylin sequentially. The number of Ki67-positive cells for each rat was counted from three consecutive sections under a microscope [17]. The experiment was independently repeated three times.

### Detection of urinary protein

The rat urine samples were collected over a 24-h period using metabolic cages on days 0, 3, 7 and 14 after injection. Thereafter, each urine sample was centrifuged for 5 min at 14,000 rpm [18]. The urine protein in the supernatant was detected using an urine protein assay kit (Pierce, Rockford, IL, USA) according to specific instructions. The experiment was independently repeated three times.

### Culture and transfection of mesangial cells

The rat mesangial cell line was commercially provided by the American Type Culture Collection (ATCC, Rockville, MD, USA) and maintained in the RPMI-1640 medium supplemented with 10% fetal bovine serum (FBS) at 37 °C and 5% CO_2_ conditions.

After reaching 85% confluence, mesangial cells were harvested after trypsinization. Then, cells were prepared into the single cell suspension with serum-free RPMI-1640 medium at a cell concentration of 1 × 10^5^ cells/mL. Thereafter, 1 mL cell suspension was added into each well of the 6-well plates. shRNA targeting Ntrk1 and corresponding negative control, pcDNA-Ntrk1 vector and empty vector were purchased from Genechem (Shanghai, China), and transfected into mesangial cells using Lipofectamine 2000 (Thermo Fisher Scientific, Waltham, MA, USA) in strict accordance with specific instructions. Cells were sequentially grouped as shNtrk1 group, shNC group, Ntrk1 group and Vector group. After 8 h of transfection, cells were cultured in RPMI-1640 medium supplemented with 10% FBS for 48 h.

### Treatment of mesangial cells with p38 inhibitor and ERK inhibitor

p38 inhibitor (SB202190) and ERK inhibitor (PD98059) were utilized respectively to treat mesangial cells. Briefly, the pcDNA-Ntrk1 vector-transfected mesangial cells were cultured with RPMI-1640 medium containing 10% FBS and 1 µM [19] SB202190 (VB2726-10, AmyJet Scientific, Wuhan, China) or 10 µM [20] PD98059 (S1805, Beyotime, Shanghai, China) at 37 °C and 5% CO_2_ conditions.

### Cell counting kit-8 (CCK-8) assay

The cell viability of mesangial cells was investigated using the CCK-8 assay. In brief, mesangial cells were prepared into the single cell suspension with RPMI-1640 medium containing 10% FBS at a cell density of 1 × 10^5^ cells/mL. Thereafter, 100 μL cell suspension was seeded into each well of the 96-well plates. Three replicate wells were set for each group, and the plates were placed under 37 °C and 5% CO_2_ conditions for 48 h. Subsequently, 10 μL CCK-8 reagent (Beyotime, Shanghai, China) was added into each well to incubate cells for 4 h at 37 °C. The optical density (OD) of each well was measured at 450 nm using a microplate reader. The experiment was independently repeated thrice.

### The 5-Ethynyl-2′-deoxyuridine (EdU) assay

The proliferation of mesangial cells was investigated by the EdU assay. Cells were dispersed into RPMI-1640 medium containing 10% FBS (1 × 10^5^ cells/mL) and seeded in the 6-well plates for 48 h under 37 °C and 5% CO_2_ conditions. Thereafter, 1 mL cell suspension was added into each well. Thereafter, cell proliferation was investigated using an EdU immunofluorescence assay in line with specific instructions (Beyotime, Shanghai, China). EdU-positive cells presented with red fluorescence, meanwhile, the nucleus was stained with blue fluorescence by using 4',6-diamidino-2-phenylindole (DAPI). The EdU-positive cells were counted under a fluorescence microscope from five random non-overlapping fields of view (FOVs). The experiment was independently repeated three times.

### Quantitative real-time polymerase chain reaction (qRT-PCR)

Total RNAs in glomeruli and mesangial cells were extracted with TRIzol reagent (Solarbio, Beijing, China) strictly in line with specific instructions. Thereafter, 3 µg total RNA samples were collected for the synthesis of cDNA template by reverse transcription using a reverse transcription kit (Promega, Madison, WI, USA). qRT-PCR was carried out using SYBR Select Master Mix (Life Technologies, California, USA) and an RT-PCR detection system (Applied Biosystems, Foster City, CA, USA). The reaction procedure was as follows: 40 cycles of denaturation at 95 °C for 30 s, annealing at 55 °C for 30 s, and extension at 72 °C for 60 s. The following primers were used, for IFN-γ, sense: 5'-ACAACCCACAGATCCAGC-3', antisense: 5'-TCAGCACCGACTCCTTTT-3'; for TNF-α, sense: 5'-CCACGCTCTTCTGTCTACTG-3', antisense: 5'-GGGAACTTCTCCTCCTTGTT-3'; for IL-6, sense: 5'-GGAGTTCCGTTTCTACCT-3', antisense: 5'-CTCTGGCTTTGTCTTTCT-3'; for Ntrk1, sense: 5′-GGACAACCCTTTCGAGTTCA-3′, antisense: 5′-GTGGTGAACACAGGCATCAC-3′; for β-actin (control), sense: 5′-AGCGAGCATCCCCCAAAGTT-3′, antisense: 5′-GGGCACGAAGGCTCATCATT-3′. The relative expression of the other mRNAs was calculated by the 2^−ΔΔCt^ method. The experiment was independently repeated three times.

### Western-blot (WB) assay

Glomeruli and mesangial cells were lysed with RIPA buffer on ice for 30 min. The lysate was centrifuged at 15,000 rpm and 4 °C for 30 min. Thereafter, the supernatant was harvested and the concentration of total protein was detected with the BCA Protein Assay Kit (Beyotime, Shanghai, China). Then, 30 μg of each protein sample was collected, boiled for 5 min, and separated with 10% sodium dodecyl sulfate polyacrylamide gel electrophoresis (SDS-PAGE). Afterwards, the separated protein was transferred onto the polyvinylidene fluoride (PVDF) membrane, which was later blocked with skimmed milk (5%) for 1 h at room temperature. Protein was later probed with primary antibodies (1:1000) overnight at 4 °C, including rabbit anti-Ntrk1 (1:1000, PAB12224, AmyJet Scientific, Wuhan, China), anti-STAT3 (1:1000, ab226942, Abcam, Shanghai, China), anti-pSTAT3 (1:1000, BY-1658R, Kemin Biotechnology, Shanghai, China), anti-p38 (1:1000, ab47363, Abcam, Shanghai, China), anti-pp38 (1:1000, YB063, Yubo Biotechnology, Shanghai, China), anti-ERK (1:1000, ab17942, Abcam, Shanghai, China), anti-pERK (1:1000, HK10249, Hushi Pharmaceutical Technology, Shanghai, China), anti-AKT (1:1000, ab89402, Abcam, Shanghai, China), anti-pAKT (1:1000, A-ABV10202, AmyJet Scientific, Wuhan, China), anti-JNK (1:1000, A-AP51547, AmyJet Scientific, Wuhan, China), anti-pJNK (1:1000, K009325P, Solarbio, Beijing, China) and anti-β-actin (1:1000, ab8227, Abcam, Shanghai, China). Subsequently, the PVDF membrane was washed twice with Tris-buffered saline containing 0.1% Tween 20 buffer, and further incubated with HRP-conjugated goat anti-rabbit secondary antibodies (1:2000, ab6721, Abcam, Shanghai, China) for 2 h at room temperature. The protein bands were then developed with the enhanced chemiluminescence (ECL) substrate (Beyotime, Shanghai, China) in the LI-COR imaging system (LI-COR Biosciences, Lincoln, NE, USA). Finally, the densitometry analysis of protein blots was conducted by ImageJ analysis system (NIH, Bethesda, MD, USA). The experiment was independently repeated thrice.

### Statistical analysis

All data were obtained from three independent repeated experiments and expressed as mean ± standard deviation (SD). SPSS19.0 software was adopted for statistical analysis. Significant differences between two groups were analyzed by two-tailed paired Student's t-test. One-way analysis of variance (ANOVA) and post-hoc Tukey's test were used to analyze the differences among multiple groups. *P* < 0.05 was considered statistically significant.

## Results

### Ntrk1 was over-expressed in glomeruli of MsPGN rats

**Fig. 1:**
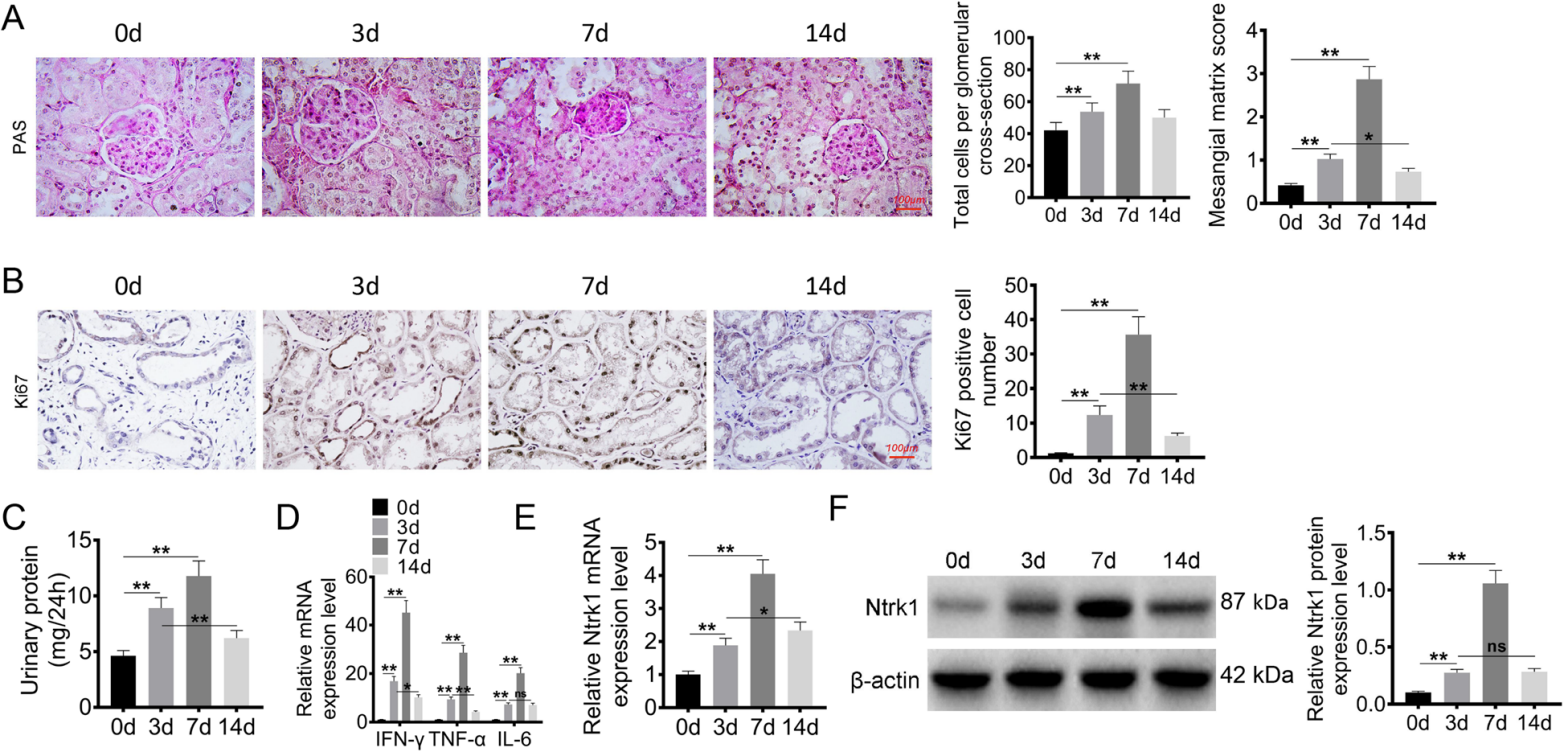
Ntrk1 was overexpressed in glomerulus of MsPGN rat. **A** PAS staining was performed on kidney tissues to detect the glomerulus injury. Total cells per glomerulus cross-section and mesangial matrix score was assessed. **B** IHC was conducted on kidney tissues to detect Ki67 positive cells. **C** Urine protein of MsPGN rat was researched. **D** Inflammatory factors expression in glomerulus was investigated using qRT-PCR, including IFN-γ, TNF-α and IL-6. **E** Ntrk1 mRNA expression in glomerulus was investigated by qRT-PCR. **F** Ntrk1 protein expression in glomerulus was assessed by Western blot. * *P* < 0.05. ** *P* < 0.01. ns, the difference is not statistically significant

PAS staining was performed to detect the glomerulus injury in MsPGN rats. On day 3 and day 7 after the injection of Thy1.1 monoclonal antibody, the mesangial cells proliferated. Relative to day 0, the total cell number per glomerulus cross-section increased on day 3 and day 7 (*P* < 0.01), which reached a peak on day 7. On day 14, the proliferation of mesangial cells declined. The difference in the total cell number per glomerulus cross-section between day 3 and day 14 was not significant. In addition, the mesangial matrix score was evaluated to assess glomerulus injury. Compared with day 0, the mesangial matrix score elevated on day 3 and day 7 (*P* < 0.01) and reached its peak on day 7. However, the mesangial matrix score decreased on day 14 compared with that on day 3 (*P* < 0.05) (Fig. 1A). IHC results exhibited more Ki67-positive cells in renal tubule on day 3 and day 7 after the injection of Thy1.1 monoclonal antibody (*P* < 0.01), which reached a peak on day 7. The Ki67-positive cell number decreased significantly on day 14, which was lower than that on day 3 (*P* < 0.01) (Fig. 1B).

After the injection of Thy1.1 monoclonal antibody, the urine protein level of MsPGN rats increased on day 3 and day 7 relative to that on day 0 (*P* < 0.01), and reached a peak on day 7. Meanwhile, the urine protein level decreased on day 14 relative to day 3 (*P* < 0.01) (Fig. 1C). The expression of inflammatory factors in glomeruli, including IFN-γ, TNF-α and IL-6, was analyzed by qRT-PCR. Relative to day 0, the expression of IFN-γ, TNF-α and IL-6 mRNAs was up-regulated on day 3 and day 7 (*P* < 0.01), which reached a peak on day 7. The IFN-γ and TNF-α mRNA levels decreased on day 14 compared with those on day 3 (*P* < 0.05 or *P* < 0.01). No obvious difference was observed in IL-6 mRNA level between day 3 and day 14 (Fig. 1D). Additionally, Ntrk1 expression in glomeruli was detected by qRT-PCR and WB assays. As a result, higher Ntrk1 mRNA and protein expression was detected on day 3 and day 7 relative to that on day 0 (*P* < 0.01), which reached a peak on day 7. The Ntrk1 mRNA expression on day 14 increased relative to that on day 14 (*P* < 0.05). However, the difference in Ntrk1 protein expression between day 14 and day 3 was not of statistical significance (Fig. 1E, F). The above results implied that the MsPGN rat model was successfully constructed and that Ntrk1 was over-expressed in glomeruli of MsPGN rats.

### Ntrk1 promoted the viability and proliferation of mesangial cells

**Fig. 2:**
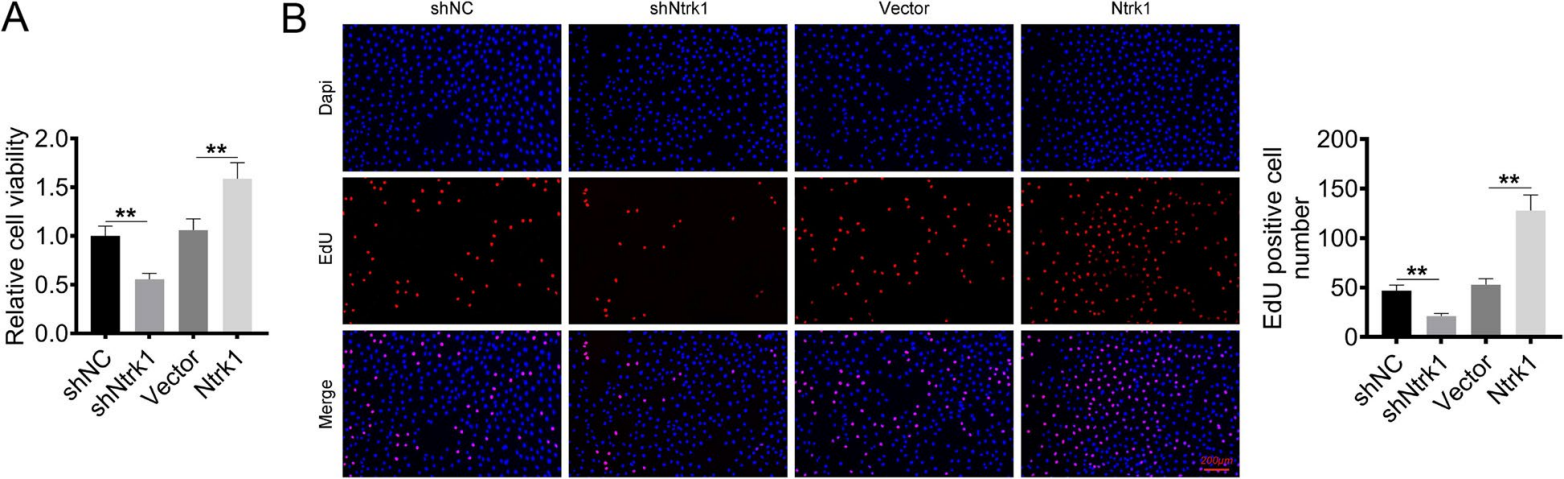
Ntrk1 promoted the viability and proliferation of mesangial cells. **A** CCK-8 assay was applied for the viability detection of mesangial cells. **B** EdU experiment was performed to explore the proliferation of mesangial cells. ** *P* < 0.01

The effect of Ntrk1 on the viability and proliferation of mesangial cells was analyzed by CCK-8 assay and EdU assay, respectively. As presented in Fig. 2A, mesangial cells of shNtrk1 group exhibited lower viability than that of shNC group (*P* < 0.01). Conversely, higher viability of mesangial cells was observed in Ntrk1 group relative to Vector group (*P* < 0.01). Simultaneously, less EdU-positive cells were detected in mesangial cells of shNtrk1 group compared with shNC group (*P* < 0.01). However, in comparison with Vector group, the number of EdU-positive cells in Ntrk1 group increased (*P* < 0.01) (Fig. 2B). Thus, Ntrk1 up-regulation promoted the viability and proliferation of mesangial cells, whereas Ntrk1 down-regulation had the opposite effect.

### Ntrk1 enhanced the expression of pro-inflammatory factors in mesangial cells

**Fig. 3:**
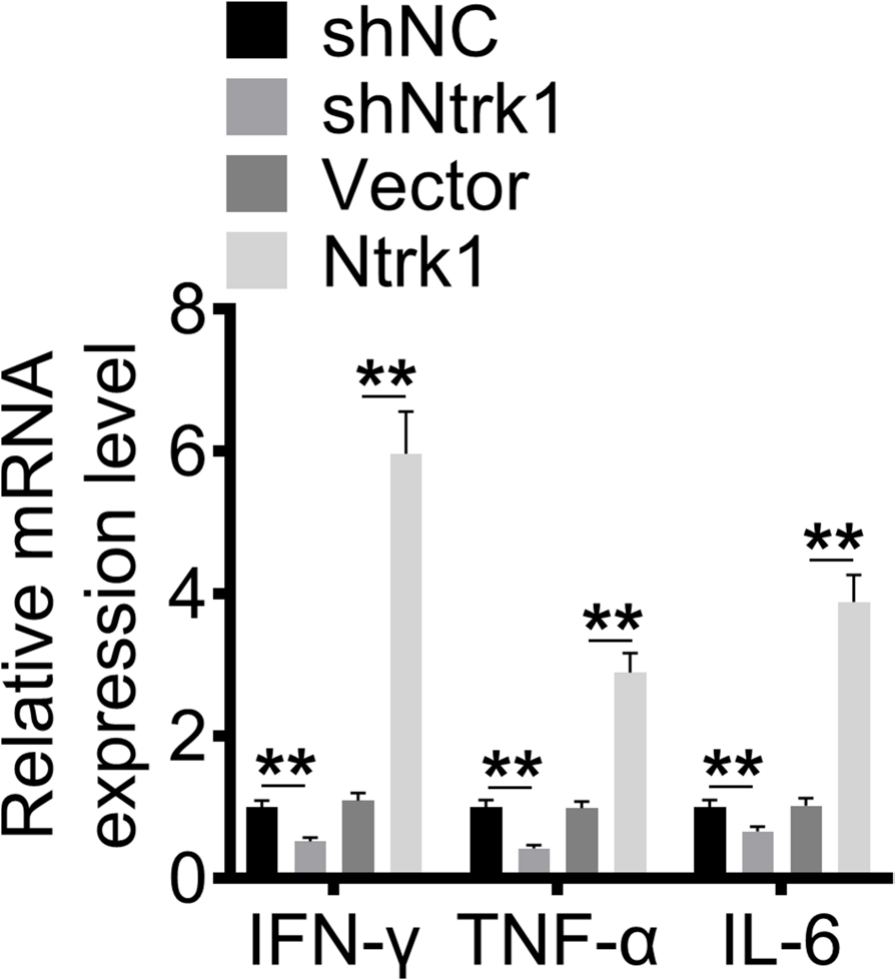
Ntrk1 enhanced the expression of pro-inflammatory factors in mesangial cells. qRT-PCR was performed to investigate the expression of pro-inflammatory factors in mesangial cells. ** *P* < 0.01

The effect of Ntrk1 on the mRNA expression of pro-inflammatory factors (including IFN-γ, TNF-α and IL-6) in mesangial cells was analyzed by qRT-PCR. As a result, lower mRNA expression of IFN-γ, TNF-α and IL-6 was observed in mesangial cells of shNtrk1 group relative to shNC group (*P* < 0.01). On the contrary, mesangial cells of Ntrk1 group showed higher mRNA expression of IFN-γ, TNF-α and IL-6 than that of Vector group (*P* < 0.01) (Fig. 3). Therefore, Ntrk1 overexpression enhanced the expression of pro-inflammatory factors in mesangial cells, whereas Ntrk1 knockdown had the opposite effect.

### Ntrk1 promoted the activation of the STAT3, p38 and ERK signaling pathways in mesangial cells

**Fig. 4:**
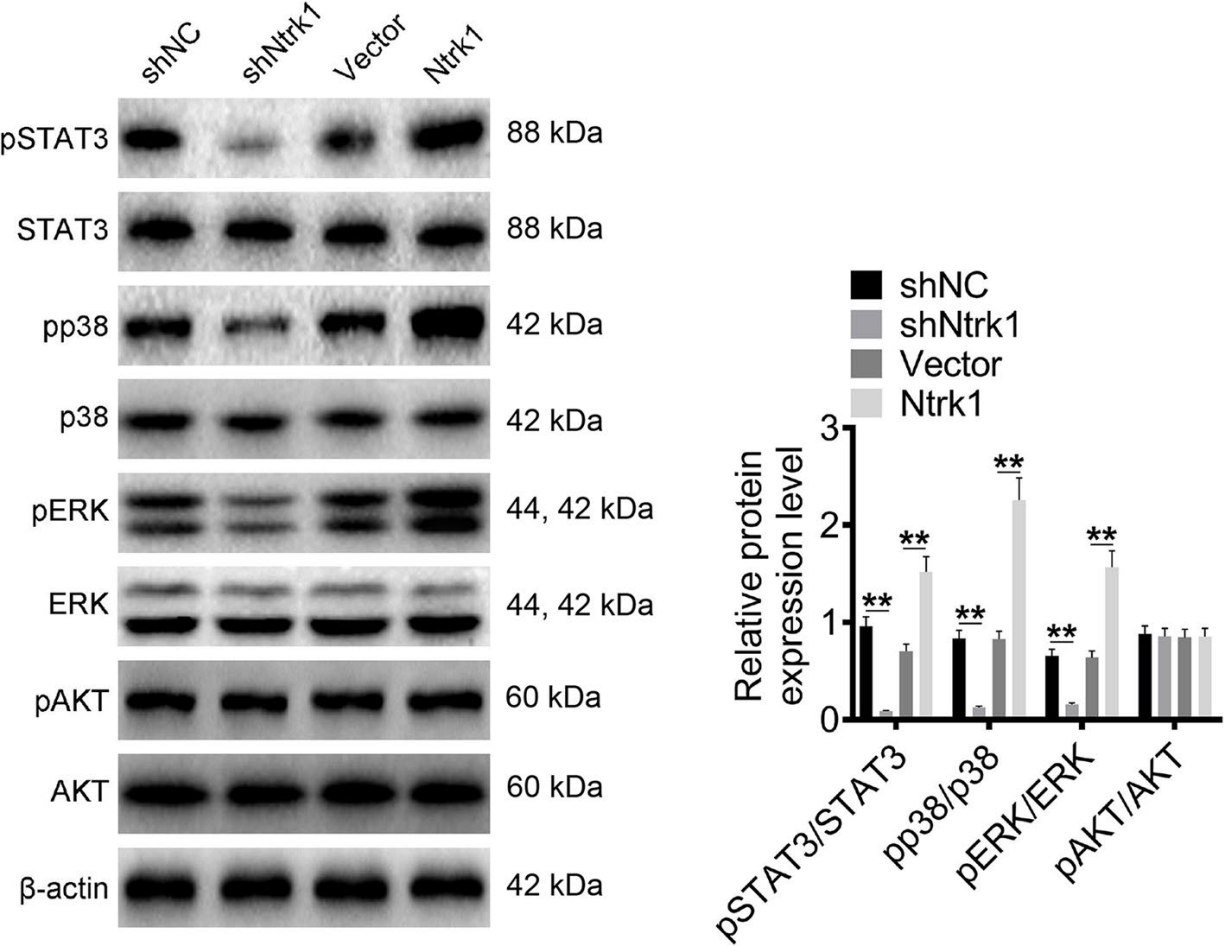
Ntrk1 promoted the activation of STAT3, p38 and ERK signaling pathways in mesangial cells. Western blot was applied for the activity detection of the STAT3, p38, ERK and AKT signaling pathways. ** *P* < 0.01

WB assay was carried out to analyze the effect of Ntrk1 on the activation of STAT3, p38, ERK, AKT and JNK signaling pathways. The results exhibited that, the expression of pSTAT3/STAT3, pp38/p38 and pERK/ERK proteins decreased in mesangial cells of shNtrk1 group relative to shNC group (*P* < 0.01). Oppositely, the expression of pSTAT3/STAT3, pp38/p38 and pERK/ERK proteins was higher in mesangial cells of Ntrk1 group than that of Vector group (*P* < 0.01). Notably, Ntrk1 knockdown or overexpression did not significantly affect the expression of pAKT/AKT and pJNK/JNK proteins (Fig. 4, Figure S1A). Hence, Ntrk1 promoted the activation of the STAT3, p38 and ERK signaling pathways in mesangial cells.

### Ntrk1 knockdown alleviated glomerulus injury in MsPGN rats

**Fig. 5:**
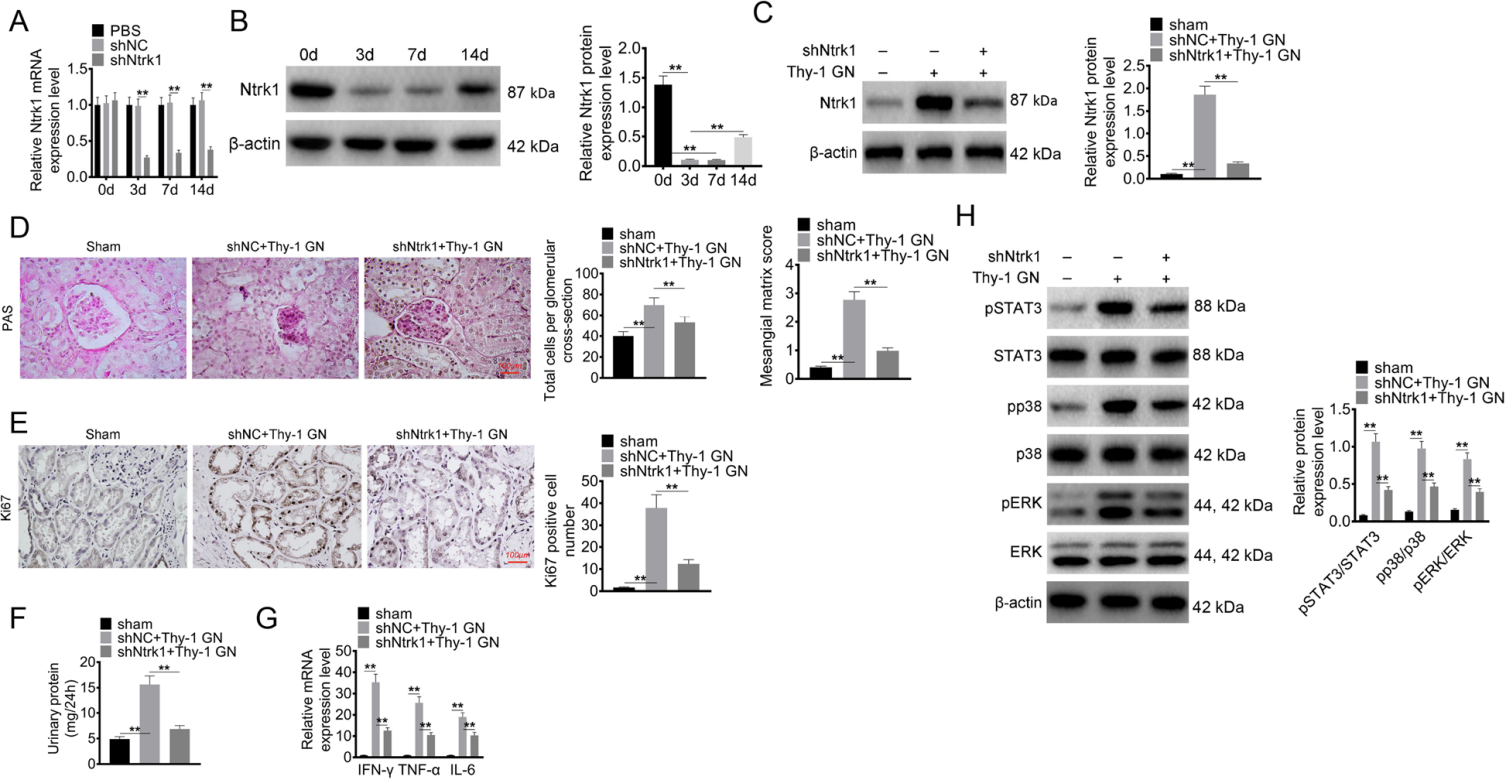
Ntrk1 knockdown alleviated glomerulus injury in MsPGN rat. **A** Ntrk1 mRNA expression in glomerulus of rats in the three groups was explored using qRT-PCR. **B** Ntrk1 protein expression in glomerulus of rats in shNtrk1 group was researched by Western blot. **C** The Ntrk1 protein expression in glomerulus of rats in each group was detected after 7 days of Thy1.1 monoclonal antibody injection. **D** After 7 days of Thy1.1 monoclonal antibody injection, PAS staining was performed on kidney tissues to research the total cells per glomerulus cross-section and mesangial matrix score. **E** IHC was conducted on kidney tissues to detect Ki67 positive cells on day 7 of Thy1.1 monoclonal antibody injection. **F** On day 7 of Thy1.1 monoclonal antibody injection, the urine of rats in each group was collected to assess the level of urinary protein. **G** On day 7 of Thy1.1 monoclonal antibody injection, the mRNA expression of inflammatory factors (IFN-γ, TNF-α and IL-6) in glomerulus of rats in each group was evaluated using qRT-PCR. **H** After 7 days of Thy1.1 monoclonal antibody injection, the activation of STAT3, p38 and ERK signaling pathways in glomerulus was explored by Western blot. ** *P* < 0.01

The lentivirus was injected via the tail vein to construct the Ntrk1 knockdown rat model. Thereafter, Ntrk1 mRNA expression in glomeruli was analyzed by qRT-PCR. As a result, the difference in Ntrk1 mRNA expression on day 0 was not significant among PBS group, shNC group and shNtrk1 group. Notably, lower Ntrk1 mRNA expression in glomeruli was found in rats of shNtrk1 group relative to shNC group (*P* < 0.01) (Fig. 5A). Ntrk1 protein expression in glomeruli of rats from shNtrk1 group was analyzed by WB assay. Besides, lower Ntrk1 protein expression in glomeruli was found on day 3 and day 7 relative to day 0 (*P* < 0.01). However, higher Ntrk1 protein expression on day 14 was observed compared with day 3 (*P* < 0.01) (Fig. 5B).

The glomerulus injury of Ntrk1 knockdown rat model was evaluated on day 7 after the injection of Thy1.1 monoclonal antibody. In comparison with Sham group and shNtrk1 + Thy-1 GN group, rats of shNC + Thy-1 GN group showed higher Ntrk1 protein expression in glomeruli (*P* < 0.01) (Fig. 5C). As revealed by PAS staining, the total cells per glomerulus cross-section and mesangial matrix score were higher in rats of shNC + Thy-1 GN group than those of Sham group and shNtrk1 + Thy-1 GN group (*P* < 0.01) (Fig. 5D). According to IHC staining, more Ki67-positive cells were found in renal tubule of shNC + Thy-1 GN group than those of Sham group and shNtrk1 + Thy-1 GN group (*P* < 0.01) (Fig. 5E). Furthermore, the urinary protein level in glomeruli of shNC + Thy-1 GN group was higher than those of Sham group and shNtrk1 + Thy-1 GN group (*P* < 0.01) (Fig. 5F). Compared with Sham group and shNtrk1 + Thy-1 GN group, higher mRNA expression of IFN-γ, TNF-α and IL-6 was observed in glomeruli of shNC + Thy-1 GN group (*P* < 0.01) (Fig. 5G). In comparison with Sham group and shNtrk1 + Thy-1 GN group, the expression of pSTAT3/STAT3, pp38/p38 and pERK/ERK proteins in glomeruli of shNC + Thy-1 GN group significantly increased (*P* < 0.01) (Fig. 5H). These results indicated that Ntrk1 knockdown alleviated glomerulus injury in MsPGN rats.

### Treatment with p38 inhibitor and ERK inhibitor reversed the effect of Ntrk1 on promoting the viability, proliferation and inflammatory response of mesangial cells

**Fig. 6:**
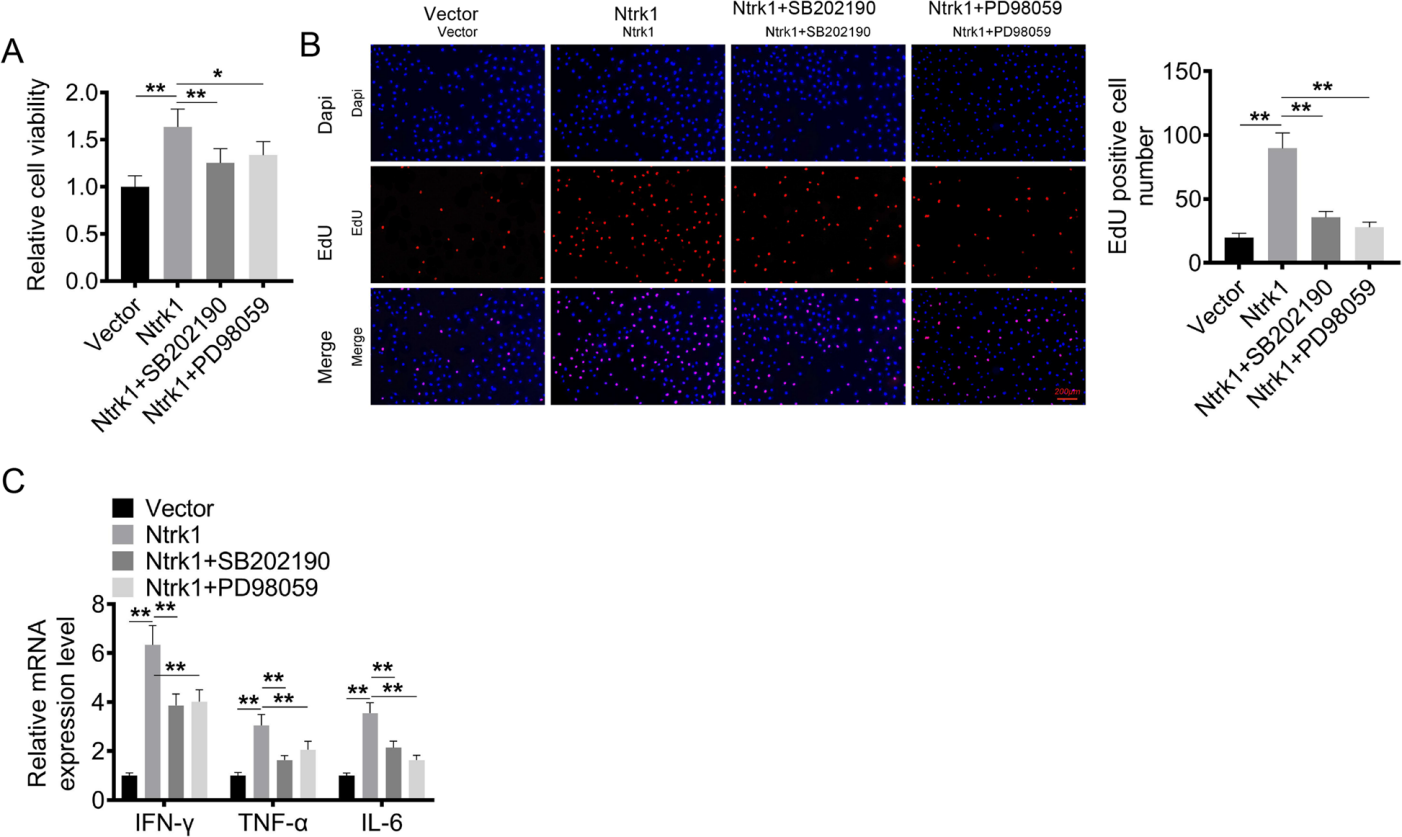
Ntrk1 might promote the viability, proliferation and inflammatory response of mesangial cells by activating the p38 and ERK signaling pathways. **A** CCK-8 assay to assess the viability of mesangial cells. **B** EdU experiment to evaluate the proliferation of mesangial cells. **C** qRT-PCR to monitor the pro-inflammatory factors (IFN-γ, TNF-α and IL-6) expression in mesangial cells. * *P* < 0.05. ** *P* < 0.01

In this study, p38 inhibitor (SB202190) and ERK inhibitor (PD98059) were utilized to treat the Ntrk1-overexpressed mesangial cells. As shown in Fig. 6A, B and C, mesangial cells of Ntrk1 group exhibited higher viability, more EdU-positive cells and higher mRNA expression of IFN-γ, TNF-α and IL-6 than that of Vector group (*P* < 0.01). Intriguingly, compared with Ntrk1 group, mesangial cells of Ntrk1 + SB202190 group and Ntrk1 + PD98059 group showed lower viability, less EdU-positive cells and lower mRNA expression of IFN-γ, TNF-α and IL-6 (*P* < 0.05 or *P* < 0.01). Therefore, treatment with p38 inhibitor and ERK inhibitor reversed the effect of Ntrk1 on promoting the viability, proliferation and inflammatory response of mesangial cells. These results indicated that Ntrk1 might promote the viability, proliferation and inflammatory response of mesangial cells by activating the p38 and ERK signaling pathways.

## Discussion

As the most active cells in the glomerulus, the proliferation of mesangial cells plays an important role in the development of MsPGN. Mesangial cells can respond to multiple external stimuli (such as macromolecular substances, immune complexes and hypoxia) via the proliferative reaction [2, 21]. The main pathological feature of MsPGN includes the proliferation and expansion of mesangial cells [22]. Hence, inhibiting mesangial cell proliferation is one of the main strategies for treating MsPGN. Previous study has reported that the injection of Thy1.1 monoclonal antibody through tail vein can successfully establish the rat MsPGN model [11]. Therefore, this work constructed the rat MsPGN model through single injection of Thy1.1 monoclonal antibody (2.5 mg/kg) via the tail vein. On day 7 after the injection of Thy1.1 monoclonal antibody, mesangial cell proliferation and glomerulus inflammatory response reached a peak. Importantly, Ntrk1 mRNA and protein expression was abnormally up-regulated in the glomeruli of MsPGN rats. As revealed by studies in-vivo and in-vitro, Ntrk1 knockdown inhibited the proliferation of mesangial cells and the expression of pro-inflammatory factors (including IFN-γ, TNF-α and IL-6) in glomeruli. Meanwhile, Ntrk1 knockdown reduced the urinary protein level in MsPGN rats. As far as we know, this is the first time that Ntrk1 is identified to promote the development of MsPGN by enhancing mesangial cell proliferation and inflammatory response.

After Ntrk1 knockdown, the activity of the STAT3 signaling pathway was reduced in glomeruli of MsPGN rats and mesangial cells. Meanwhile, Ntrk1 knockdown alleviated glomerulus injury in MsPGN rats. Previous study has reported that the activation of STAT3 signaling pathway is an important factor to induce the progression of glomerulonephritis. The level of phosphorylated STAT3 (pSTAT3) is consistent with the peak of mesangial cell proliferation in glomeruli. Interestingly, the expression of pSTAT3 decreased with the attenuation of glomerular proliferation and sclerosis. Thus, the activation of the STAT3 signaling pathway enhances the progression of glomerulus sclerosis in glomerulonephritis [23]. Meanwhile, the activation of the STAT3 signaling pathway is demonstrated to be critical for the development of crescentic glomerulonephritis, which exacerbates the proliferation of glomerular cells and the damage of kidney [24]. Therefore, in this work, Ntrk1 might promote the viability, proliferation and inflammatory response of mesangial cells by stimulating the activation of the STAT3 signaling pathway.

p38 is one of the main members of the MAPK signaling pathway, and its activation can lead to the proliferation of mesangial cells in diabetic nephropathy [25]. Zhu et al*.* [26] revealed that the activation of the p38 signaling pathway was necessary for the development of MsPGN in rats. As another member of the MAPK family, ERK is the core of the signaling network that regulates cell growth and division [27]. Leonard et al*.* [28] illustrated that the activation of p38 and ERK signaling pathways is crucial for the production of IL-6 in mesangial regions of glomeruli. Simultaneously, the activation of ERK signaling pathway is demonstrated to aggravate the proliferation of mesangial cells in the rat MsPGN model [29]. In lupus nephritis, the expression of phosphorylated ERK (pERK) is reduced by miR-155, which significantly inhibits the proliferation of mesangial cells [30]. The activation of ERK signaling pathway is reported to be involved in the inflammatory response. Sun et al*.* [31] found that the inactivation of ERK signaling pathway alleviated the inflammatory response. In this study, it was demonstrated that Ntrk1 knockdown suppressed the phosphorylation of p38 and ERK. On the opposite, Ntrk1 up-regulation promoted the phosphorylation of p38 and ERK. More interestingly, treatment with p38 inhibitor and ERK inhibitor reversed the effect of Ntrk1 on promoting the viability, proliferation and inflammatory response of mesangial cells. As suggested by in-vivo study, Ntrk1 knockdown alleviated glomerulus injury in MsPGN rats, and suppressed the activity of p38 and ERK signaling pathways. Taken together, Ntrk1 might promote the viability, proliferation and inflammatory response of mesangial cells by stimulating the activation of p38 and ERK signaling pathways.

In previous studies, the AKT signaling pathway has been found to be activated in mesangial cells, which represents a key mechanism for promoting the proliferation and inflammatory response of mesangial cells in rats with MsPGN [2, 21, 32]. Moreover, JNK is a crucial member of the MAPK signaling pathway [33]. The repressed activity of the JNK signaling pathway is implied to attenuate the proliferation of mesangial cells, so as to relieve renal lesions in IgA nephropathy [34]. Intriguingly, this study implied that Ntrk1 did not significantly affect the activation of AKT and JNK signaling pathways. The underlying mechanism of this phenomenon will be investigated in our future research.

Certain limitations should be noted in this paper. Previous studies have implied that there is crosstalk between the STAT3 and MAPK signaling pathways [35–37]. Therefore, it will be better to verify whether there is an upstream and downstream regulatory relationship between the STAT3 and MAPK signaling pathways. Moreover, this paper indicated that Ntrk1 overexpression or knockdown did not obviously affect the activation of AKT and JNK signaling pathways in mesangial cells. It should be better to conduct a more in-depth study on the underlying mechanism of this phenomenon. However, due to the limitations of laboratory conditions, these issues cannot be currently verified. We will devote ourselves to investigating these interesting issues in the future research.

In summary, this paper established a rat MsPGN model and explored the effect of Ntrk1 on the proliferation and inflammatory response of mesangial cells. The results revealed that Ntrk1 was abnormally over-expressed in glomeruli of MsPGN rats. Ntrk1 knockdown reduced the proliferation and inflammation of mesangial cells, whereas Ntrk1 overexpression showed the opposite effect. In terms of the underlyign mechanism, Ntrk1 might promote the proliferation and inflammation of mesangial cells via activating the STAT3, p38 and ERK signaling pathways. Thus, Ntrk1 might be a promising therapeutic target for MsPGN.

## Supplementary Information

**Additional file 1: Figure S1.**  

## Data Availability

The datasets used and/or analysed during the current study are available from the corresponding author on reasonable request.

## Abbreviations

MsPGN: Mesangial proliferative glomerulonephritis
Ntrk1: Neurotrophic tropomyosin receptor kinase 1
Trk: Tyrosine receptor kinase
TrkA: Tyrosine receptor kinase A
