# Supplementary figures and images for: Ntrk1 promotes mesangial cell proliferation and inflammation in rat glomerulonephritis model by activating the STAT3 and p38/ERK MAPK signaling pathways
Source: BMC Nephrol. 2022 Dec 28;23:413. doi: 10.1186/s12882-022-03001-4 (PMC9795628; doi:10.1186/s12882-022-03001-4)

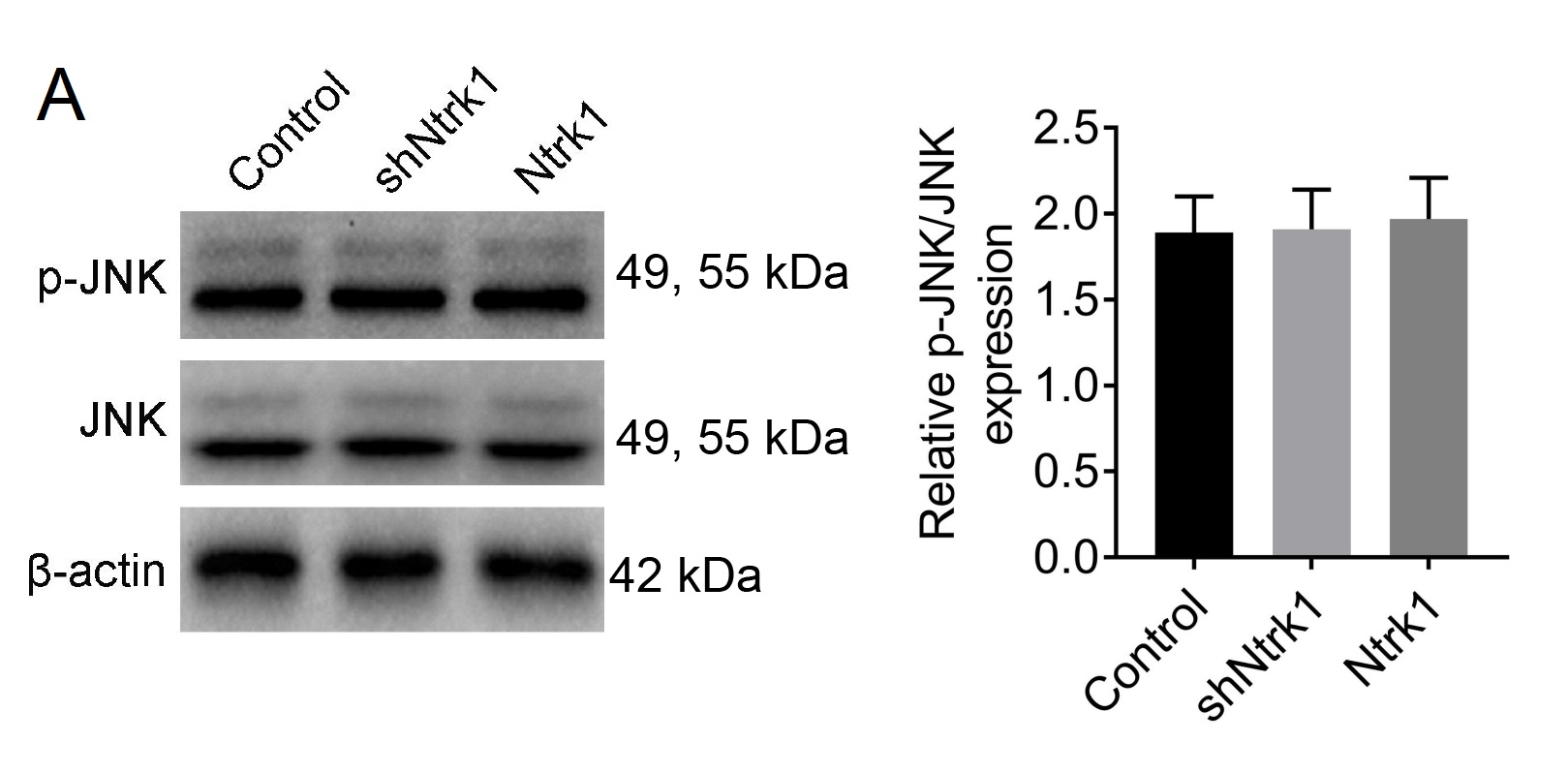

Supplement: Supplementary file 1 — Additional file 1: Figure S1. [file 12882_2022_3001_MOESM1_ESM.jpg]
